# Optimizing immunogenicity and product presentation of a SARS-CoV-2 subunit vaccine composition: effects of delivery route, heterologous regimens with self-amplifying RNA vaccines, and lyophilization

**DOI:** 10.3389/fimmu.2024.1480976

**Published:** 2024-12-16

**Authors:** William R. Lykins, Jeroen Pollet, Jessica A. White, Brian Keegan, Leroy Versteeg, Ulrich Strych, Wen-Hsiang Chen, Raodoh Mohamath, Gabi Ramer-Denisoff, Sierra Reed, Christina Renshaw, Samuel Beaver, Alana Gerhardt, Emily A. Voigt, Mark A. Tomai, Robert Sitrin, Robert K. M. Choy, Frederick J. Cassels, Peter J. Hotez, Maria Elena Bottazzi, Christopher B. Fox

**Affiliations:** ^1^ Access to Advanced Health Institute, Seattle, WA, United States; ^2^ Texas Children’s Hospital Center for Vaccine Development, Baylor College of Medicine, Houston, TX, United States; ^3^ Department of Pediatrics, National School of Tropical Medicine, Baylor College of Medicine, Houston, TX, United States; ^4^ PATH, Seattle, WA, United States; ^5^ 3M Health Care, St. Paul, MN, United States; ^6^ Department of Biology, Baylor University, Waco, TX, United States; ^7^ Department of Global Health, University of Washington, Seattle, WA, United States

**Keywords:** RNA vaccine, heterologous vaccine, intranasal vaccine, receptor binding domain, adjuvant formulation, vaccine development, lyophilized vaccine

## Abstract

**Introduction:**

Dozens of vaccines have been approved or authorized internationally in response to the ongoing SARS-CoV-2 pandemic, covering a range of modalities and routes of delivery. For example, mucosal delivery of vaccines via the intranasal (i.n.) route has been shown to improve protective mucosal responses in comparison to intramuscular (i.m.) delivery. As we gain knowledge of the limitations of existing vaccines, it is of interest to understand if changes in product presentation or combinations of multiple vaccine modalities can further improve immunological outcomes.

**Methods:**

We investigated a commercial-stage SARS-CoV-2 receptor binding domain (RBD) antigen adjuvanted with a clinical-stage TLR-7/8 agonist (3M-052) formulated on aluminum oxyhydroxide (Alum). In a murine immunogenicity model, we compared i.n. and i.m. dosing of the RBD-3M-052-Alum vaccine. We measured the magnitude of antibody responses in serum and lungs, the antibody-secreting cell populations in bone marrow, and antigen-specific cytokine-secreting splenocyte populations. Similarly, we compared different heterologous and homologous prime-boost regimens using the RBD-3M-052-Alum vaccine and a clinical-stage self-amplifying RNA (saRNA) vaccine formulated on a nanostructured lipid carrier (NLC) using the i.m. route alone. Finally, we developed a lyophilized presentation of the RBD-3M-052-Alum vaccine and compared it to the liquid presentation and a heterologous regimen including a previously characterized lyophilized form of the saRNA-NLC vaccine.

**Results and discussion:**

We demonstrate that i.n. dosing of the RBD-3M-052-Alum vaccine increased IgA titers in the lung by more than 1.5 logs, but induced serum IgG titers 0.8 logs lower, in comparison to i.m. dosing of the same vaccine. We also show that the homologous prime-boost RBD-3M-052-Alum regimen led to the highest serum IgG and bronchial IgA titers, whereas the homologous saRNA-NLC regimen led to the highest splenocyte interferon-γ response. We found that priming with the saRNA-NLC vaccine and boosting with the RBD-3M-052-Alum vaccine led to the most desirable immune outcome of all regimens tested. Finally, we show that the lyophilized RBD-3M-052-Alum vaccine retained its immunological characteristics. Our results demonstrate that the route of delivery and the use of heterologous regimens each separately impacts the resulting immune profile, and confirm that multi-product vaccine regimens can be developed with stabilized presentations in mind.

## Introduction

As SARS-CoV-2 continues to be of significant global concern, it has become evident that the choice of a specific booster or annual vaccine is primarily influenced by availability, efficacy, and compatibility with prior vaccines an individual has received (1). Moving forward, as the vaccine market continues to diversify not only in terms of disease targets but also in vaccine modalities, there is a need to not only establish if and how vaccine products interact but to also design vaccine products with those interactions in mind. Additionally, there is a need to evaluate how existing vaccine compositions perform with different presentations, such as needle-free delivery or thermostabilized formulations, which would simplify the logistics of global vaccination campaigns by reducing dependence on clinical staff and cold-chain transport, ultimately reducing cost (2, 3).

For vaccine development against respiratory pathogens, such as SARS-CoV-2, there is sometimes a tradeoff between the development of a conventional intramuscular (i.m.) formulation, which is capable of generating a systemic humoral and cellular response sufficient to limit severe disease and viremia, and the more challenging development of a mucosally delivered product (oral, sublingual, intranasal, intrapulmonary, etc.), which may be capable of generating a more robust response at the mucosal surface and subsequently preventing infection and transmission by enhancing the secretion of IgA and other mucosal immunity mediators (4–7). To date, no mucosally delivered SARS-CoV-2 vaccine products have been approved by the United States (US) Food and Drug Administration (FDA); however, multiple mucosal SARS-CoV-2 vaccine products are currently in clinical trials, and at least four have been approved for human use in China, India, Iran, and Russia, primarily composed of adenovirus vectors or adjuvanted subunit antigens (8–10). The current i.m. mRNA-based vaccines that have dominated the market in the USA and Europe are not able to induce robust mucosal immune responses but have been shown to prevent severe systemic disease (11, 12). Therefore, the characterization of existing vaccine compositions via alternate routes of administration might indicate if additional development is warranted to generate a mucosal vaccines product.

An important consideration for COVID-19 vaccines that are attempting to enter clinical trials is their immunological compatibility with both existing vaccine platforms and pre-existing immunity acquired from natural infection. As of May 2023, the CDC estimated that 87.9% of adults in the USA had received at least one COVID-19 vaccine dose, and 33.9% of vaccinated adults had received at least two doses, including one or more updated bi-valent COVID-19 vaccine booster (13). The low population uptake of new or updated annual immunizations and boosters is under investigation, but among the possible reasons is the modest impact of these vaccines on preventing infection and transmission despite their proven ability to reduce severe illness (14, 15). Therefore, emerging vaccines would benefit from testing with established vaccine platforms early on in development to ensure that they lead to productive outcomes in a heterologous prime-boost regimen. A handful of clinical trials have examined the effect that heterologous vaccine regimens have on protection against SARS-CoV-2, and several groups have explored the use of heterologous vaccine regimens in animal models using RNA and subunit vaccines (16–20). However, to our knowledge, no group has looked specifically at the use of self-amplifying RNA (saRNA) vaccines in heterologous combination with a TLR-7/8 adjuvanted subunit vaccine, both of which represent emerging vaccine technologies (21, 22).

We previously developed a SARS-CoV-2 subunit vaccine using the RBD203-N1 antigen, present in the *IndoVac* vaccine that is currently used in Indonesia (as either a primary immunization or booster) (23, 24). We adjuvanted this RBD antigen with the TLR-7/8 agonist 3M-052 formulated with aluminum oxyhydroxide (Alum) (25–29). The RBD-3M-052-Alum vaccine led to enhanced humoral responses (serum IgG and lung IgA), bone marrow-resident antibody-secreting cell populations, and serum pseudovirus-neutralizing titers compared to the unadjuvanted protein (hereafter referred to as RBD) and RBD-Alum. Here, we present the use of the RBD-3M-052-Alum vaccine as a model adjuvanted subunit vaccine to test intranasal (i.n.) delivery, heterologous combination with an RNA-based vaccine, and proof-of-concept development of a thermostable RBD-3M-052-Alum formulation. We found that the route of delivery has a measurable effect on mucosal IgA production but did not otherwise impact the observed immune phenotype. We also demonstrate that a heterologous regimen with a clinical-stage saRNA vaccine, delivered using a nanostructured lipid carrier formulation (saRNA-NLC) (30–33), improves humoral and effector cell responses compared to either homologous vaccine regimen. Finally, we demonstrate a proof-of-concept lyophilized presentation for the RBD-3M-052-Alum vaccine and show that immunogenicity is maintained following lyophilization. Our results show that the delivery route and combination regimen influence the induced immune phenotype and that suitable lyophilized presentations can be developed without detrimental impact on immunogenicity. Our study further suggests that investigation of these factors with other vaccine compositions may be merited.

## Materials and methods

### Raw materials

Recombinant wild-type (wt) (Wuhan-Hu-1) RBD203-N1 was provided by Texas Children’s Hospital Center for Vaccine Development (Houston, TX). Aluminum hydroxide (Alhydrogel 2%) was procured from Croda (Princeton, NJ; #21645-51-2). Shark squalene (#S3626), Dynasan 114 (#T5141), and Tween 80 (#1.37171) were procured from Sigma-Aldrich (St. Louis, MO). Span 60 (#TCI-S0062) and sodium citrate anhydrous (#S1986) were procured from Spectrum Chemical (New Brunswick, NJ). 1,2-distearoyl-sn-glycero-3-phospho-rac-glycerol sodium (DSPG-Na; #840465X) and 1,2-dioleoyl-3-trimethylammonium-propane chloride (DOTAP; #D-67065) were procured from Lipoid (Ludwigshafen, Germany). 0.9% (w/v) saline was produced in-house. Unless otherwise noted, all aqueous buffers were produced using Milli-Q water (MilliporeSigma, Burlington, MA). All other materials (unless otherwise noted) were acquired from Fisher Scientific (Hampton, NH).

### Self-amplifying RNA production

SARS-CoV-2 Spike protein encoding saRNA, using a Venezuelan equine encephalitis virus backbone, production and purification was performed via *in vitro* transcription as previously described (31, 32). The final saRNA product was buffer exchanged into 10 mM Tris-HCl at pH 8 prior to sterile filtration. Product purity was measured via gel electrophoresis, and saRNA content was quantified using a Quant-it RiboGreen RNA assay (Thermo Fisher Scientific). saRNA was stored at -80°C until use.

### Vaccine production and mixing

All adjuvants, formulations, and vaccines were prepared under aseptic conditions and used within an hour of mixing or reconstitution (in the case of the lyophilized product). 3M-052-AF and 3M-052-Alum were prepared as previously described (34). 3M-052-AF was manufactured by dispersing DSPG-Na and 3M-052 in chloroform at a 5.4:1 mass ratio in a round-bottom flask, then drying into a homogeneous thin film overnight via rotary evaporation. The thin film was then dispersed in Milli-Q water at 0.25 mg/mL 3M-052 via ultrasonic bath sonication at 60°C until a translucent cloudy liquid was obtained with no visible particles. The 3M-052-DSPG particles were further reduced in size using an M-110P Microfluidizer (Microfluidics, Newton, MA) at 30,000 psi to obtain 80 ± 10 nm.d particles prior to sterile filtration with a 0.8-μm/0.2-μm PES syringe filter (#4658) (Cytiva, Marlborough, MA). 3M-052-Alum was prepared by combining 3M-052-AF with Alhydrogel (2%) and diluting with ultrapure water for injection to a final concentration of 0.08 mg/mL 3M-052 and 4 mg/mL aluminum. The 3M-052-Alum mixture was mixed at room temperature on an orbital shaker for at least 10 min to ensure complete binding before the addition of the antigen. RBD aliquots were individually frozen and stored at -80°C. Prior to mixing, RBD aliquots were thawed at room temperature and diluted to 0.28 mg/mL in 0.9% w/v saline. The RBD-3M-052-Alum vaccine was mixed to achieve the concentrations outlined in **Table 1** and **Table 2** and dosed at 50 μL or 100 μL per animal as described in the figure captions using sterile 0.9% w/v saline as a diluent and stored on ice until use. All liquid subunit vaccine samples were mixed and used within 4 h of thawing the RBD.

**Table 1:** Dosing scheme.

#	Group	RBD (μg)	Formulation	Agonist
1	RBD Control (i.m.)	7	–	–
2	RBD-3M-052-Alum (i.m.)	7	Alum (100 μg)	3M-052 (2 μg)
3	RBD Control (i.n.)	7	–	–
4	RBD-3M-052-Alum (i.n.)	7	Alum (100 μg)	3M-052 (2 μg)

Animals were vaccinated on Days 0 and 21 with the indicated vaccine preparations and routes of administration. Serum and tissues were collected on Day 42. Doses of each component were given per animal per vaccination. All vaccines were prepared at 50 μL total volume. Intranasal (i.n.) groups received 25 μL per nare at each vaccination. Intramuscular (i.m.) doses were evenly split bilaterally in the rear biceps femoris at each vaccination. Alum doses are given in terms of aluminum mass.

**Table 2:** Heterologous dosing design.

Group	Prime (Day 0)	Boost (Day 21)
2x saRNA-NLC	saRNA-NLC	saRNA-NLC
2x RBD-3M-052-Alum	RBD-3M-052-Alum	RBD-3M-052-Alum
saRNA-NLC → RBD-3M-052-Alum	saRNA-NLC	RBD-3M-052-Alum
RBD-3M-052-Alum → saRNA-NLC	RBD-3M-052-Alum	saRNA-NLC

Animals were vaccinated i.m. on Days 0 and 21 with the indicated vaccine preparations for each regimen, and serum and tissues were collected on Day 42. saRNA-NLC doses contained 10 μg saRNA per dose. RBD-3M-052-Alum doses contained 7 μg RBD, 2 μg 3M-052, and 100 μg aluminum (Alum) per dose. All vaccinations were prepared in 100 μL and dosed in two 50-μL injections bilaterally in the rear biceps femoris.

NLCs were prepared as previously described (35). Briefly, squalene, DOTAP, Span 60, and Dynasan 114 were combined and allowed to fully mix at 65°C via vortexing and gentle shaking. The aqueous phase was prepared by combining Tween 80 and sodium citrate with Milli-Q water, mixing on a magnetic stir plate at room temperature until fully homogeneous. The aqueous phase was preheated to 65°C and combined with the oil phase using a high-shear mixer (Silverson, East Longmeadow, MA) to form a crude emulsion. The crude emulsion was then further reduced in size using an M-110P Microfluidizer (Microfluidics, Newton, MA) at 30,000 psi to achieve 45 ± 5 nm-diameter particles. NLCs were then sterile filtered using a 0.8-μm/0.2-μm PES syringe filter (#4658) (Cytiva, Marlborough, MA). The final composition before complexing was 3.75% squalene, 3.70% Tween 80, 3.70% Span 60, 3.0% DOTAP, 0.24% Dynasan 114, and 10 mM sodium citrate (all percentages given as w/v).

The saRNA and NLCs were complexed by mixing appropriately diluted aqueous saRNA 1:1 by volume with NLC diluted in a buffer containing 10 mM sodium citrate and 20% w/v sucrose. All vaccines were prepared at a nitrogen:phosphate (N:P) ratio of 15, representing the ratio of amine groups on the NLC DOTAP to phosphate groups on the RNA backbone, at a final saRNA concentration of 0.1 mg/mL. This complexing reaction produced a vaccine solution containing the intended dose of complexed saRNA-NLC in an isotonic 10% w/v sucrose, 5 mM sodium citrate solution (with <4 mM Tris buffer present from the bulk saRNA material). The saRNA-NLC vaccine was incubated on ice for 30 min after mixing to ensure complete complexing, then used within 4 h.

Particle sizes referenced above were measured via dynamic light scattering (DLS) using a Zetasizer Nano ZS (Malvern Panalytical, UK). Prior to particle size analysis, all samples were diluted 10- to 100-fold in Milli-Q water.

### Lyophilization procedure

Lyophilized saRNA-NLC vaccine was prepared according to the procedure previously used (31). Briefly, complexed saRNA-NLC material was prepared as described above but with 20% w/v sucrose in the final complexed vaccine as a lyoprotectant. Material was aliquoted into sterile 3-mL borosilicate glass vials with 0.5-mL fill volume. Vials were partially stoppered with 13-mm 2-leg butyl rubber lyophilization stoppers and placed directly on the shelf in a VirTis AdVantage 2.0 EL-85 (SP Industries, Warminster, PA) benchtop lyophilizer. The freezing step occurred at -50°C followed by primary drying at -30°C and 50 mTorr. Finally, the temperature was raised to 25°C at 50 mTorr for secondary drying. Upon completion of the lyophilization cycle, pressure was increased to atmospheric, and the stoppers were fully inserted into each vial using the lyophilizer’s stoppering platen. Vials were sealed with 13-mm tear-off aluminum seals after removal from the lyophilizer. Lyophilized vials were stored at 4°C until use.

Lyophilized RBD-3M-052-Alum vaccines were prepared in a similar manner to the lyophilized saRNA-NLC vaccine above. The vaccine admixture was prepared with a final composition of 0.1 mg/mL RBD, 0.03 mg/mL 3M-052, 1.4 mg/mL Alum, and 10% w/v sucrose as a lyoprotectant. The RBD-3M-052-Alum vaccine material was then aliquoted into 3-mL glass vials with a 0.5-mL fill volume and partially stoppered. Vials were placed directly on the lyophilizer shelf and lyophilized according to the same cycle parameters as above. After the lyophilization cycle was complete, vials were brought to atmospheric pressure, stoppered, and removed from the lyophilizer. Vials were sealed with tear-off aluminum seals and placed at 4°C until use in *in vivo* studies or at 25°C and 40°C for biophysical stability testing.

### Animal use and procedures

BALB/c mice were purchased from The Jackson Laboratory (Harbor, ME). Experimental groups consisted of equal numbers of 6–8-week-old male and female mice. All presented animal experiments were performed in two halves, divided evenly by group and animal sex, and vaccinations/harvests were staggered 1 week apart to reduce operator burden. Mice immunized by i.m. injection received 50 μL or 100 μL total volume (25-50 μL in each hind leg) of vaccine, and mice immunized i.n. received 50 μL total volume (25 μL per nare) as indicated on Days 0 and 21. Serum and bronchoalveolar lavage (BAL)-based assays for each study (e.g., antibody titer, pseudovirus neutralization) were performed for all animals simultaneously using frozen serum and BAL samples, respectively. Assays relying on live cells (e.g., enzyme-linked immunosorbent spot [ELISpot]) were performed at the time of tissue harvest. All animal experiments were performed in accordance with national and institutional guidelines for animal care of laboratory animals and were approved by the Bloodworks Northwest Research Institute’s Institutional Animal Care and Use Committee (Seattle, WA).

### Serum and tissue collection

**Figure 1:**
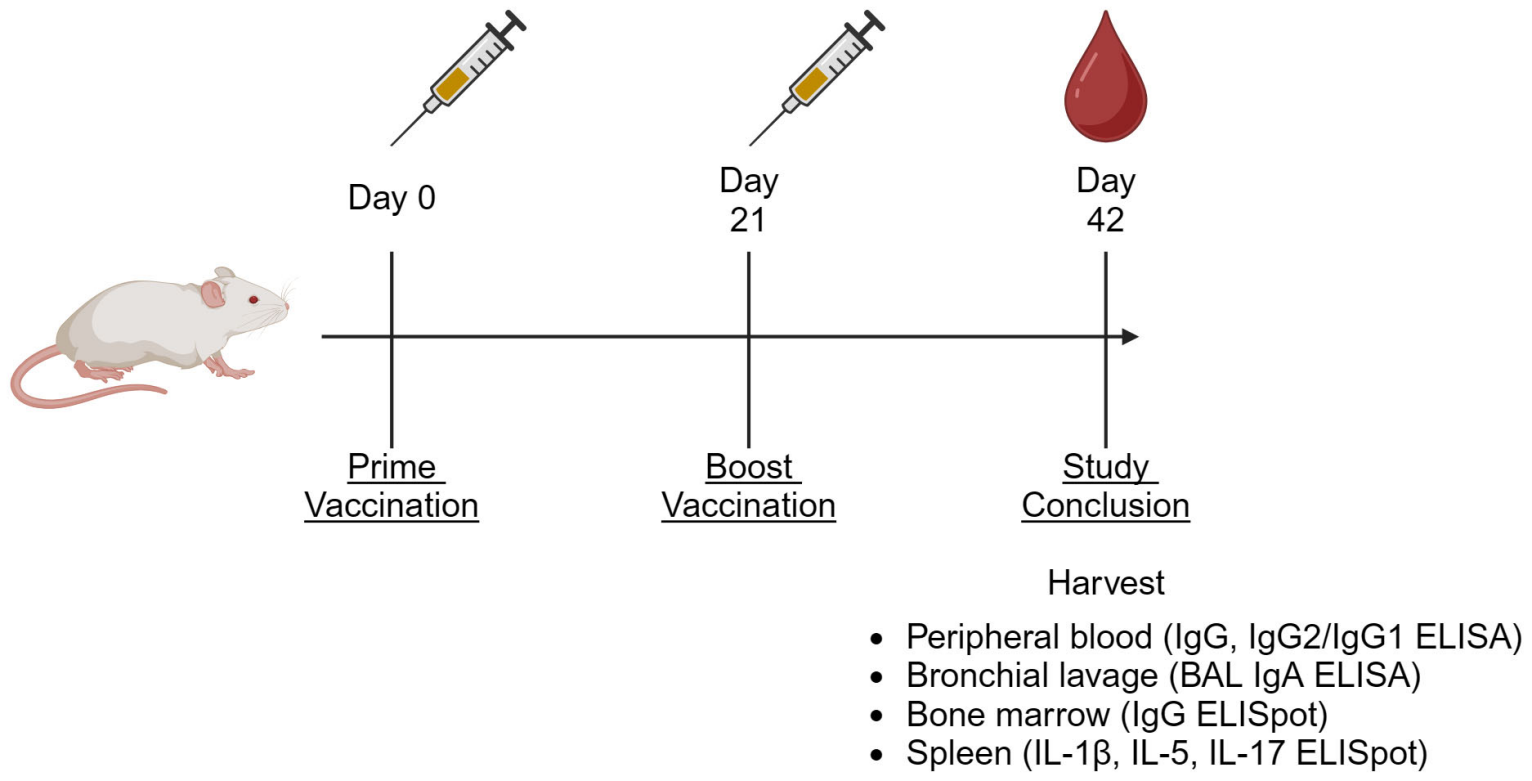
Generalized animal study diagram. For all presented *in vivo* studies, animals were vaccinated on Days 0 and 21, then euthanized on Day 42. At the termination of the study, peripheral blood, bronchial lavage (BAL), splenocytes, and bone marrow-resident cells were harvested for the indicated assays (64).

Animal procedures were performed as previously described, outlined in **Figure 1** (29). Peripheral blood was collected via cardiac puncture on Day 42. Serum was stored at -80°C until analysis. Mice were euthanized on Day 42 through carbon dioxide inhalation, followed by cervical dislocation. Serum and tissues were harvested and stored on ice immediately after euthanization. Fractionated serum and BAL samples were then stored at -80°C until analysis, and cell and tissue samples were processed on the same day as harvest.

### Serum and BAL antibody ELISA

ELISAs were performed as previously described (29). Briefly, ELISA plates were coated with RBD or full-length wt Spike protein followed by the addition of serially diluted serum or BAL fluid and subsequently HRP-conjugated detection antibodies against mouse IgG, IgG1, or IgG2a. ELISA plates were developed using a 3,3’,5,5’-tetramethylbenzidine (TMB) substrate and stopped with H_2_SO_4_. Endpoint titer was quantified by a least squares fit of A450 data to a 4-parameter sigmoidal curve, using a cutoff established by serum or BAL samples from naïve animals. Titer values that could not be quantified were set at half of the assay’s lower limit of detection.

### Bone marrow and splenocyte ELISpot

ELISpot assays were performed as previously described (29). Briefly, ELISpot plates were coated with a capture ligand: either full-length intact wt Spike protein for bone marrow IgG ELISpots, or anti-mouse IFN-γ or IL-5 for splenocyte ELISpots. Homogenized bone marrow or splenocyte tissue cell isolates were incubated on the ELISpot plates for 3-72 h. Plates were developed using HRP-conjugated detection antibodies and 3-amino-9-ethylcarbazole (AEC) substrate kits (Vector Laboratories, Newark, CA) according to the manufacturer’s protocol. Positive spots were enumerated using an automated ELISpot reader (CTL Analyzer, Cellular Technology Limited, Cleveland, OH). Data were analyzed using ImmunoSpot software (Cellular Technology Limited).

### Pseudovirus neutralization assay

SARS-CoV-2 pseudovirus neutralization assays were conducted on immunized mouse serum samples as previously described (29, 31, 36). Briefly, lentiviral pseudoviruses displaying the wt Spike protein containing a luciferase expression vector were co-incubated with serial dilutions of serum prior to addition to ACE-2 expressing HEK-293 cells. Infection inhibition curves were read via luminescence, and inhibitory IC_50_ was quantified via fitting to a 4-parameter sigmoidal curve.

### Laser diffraction particle size measurements

RBD-3M-052-Alum vaccine was characterized before and after lyophilization and storage by laser diffraction particle sizing using a Partica LA-960 (Horiba Scientific, Piscataway, NJ). Liquid or reconstituted vaccine material was loaded into the sample bath and kept in suspension using an agitator arm and circulation pump. Enough sample was loaded to bring the instrument’s laser transmittance within the target measurement range, then triplicate measurements were taken. The sample bath was flushed and refilled with distilled water between samples. The mean size calculated by the instrument software for each replicate was averaged to calculate the particle size for a given sample.

### Dynamic scanning fluorimetry

The RBD-3M-052-Alum vaccine was characterized before and after lyophilization and storage by nano differential scanning fluorimetry (nanoDSF) using a Prometheus NT.48 (NanoTemper Technologies, München, Germany) to assay antigen stability. Liquid or reconstituted vaccine material (in triplicate for each sample) was filled into high-sensitivity capillaries (NanoTemper Technologies, München, Germany), and the capillaries were sealed. The capillaries were equilibrated to 15°C in the instrument, and then a temperature melt was performed from 15°C to 95°C with a ramp rate of 0.2°C/min with the instrument exciting the samples at 295 nm and measuring emission at 330 nm and 350 nm using 30% excitation power. Using the instrument software, the melting curve of the ratio of 350:330 nm emission versus temperature was plotted, and the Tm was determined.

### Statistical analyses

Adaptive immune responses measured in vaccinated animals were log-transformed as indicated. Experimental groups were compared via a one- or two-way ANOVA followed by Holm-Sidak’s correction for multiple comparisons as indicated in figure legends. All statistical analyses were performed using GraphPad Prism 10.1.2 (San Diego, CA).

## Results

### Testing alternate routes of administration

To address the significant interest in a SARS-CoV-2 vaccine that effectively generates a mucosal immune response at the site of infection, we compared i.n. dosing of the 3M-052-Alum adjuvanted RBD vaccine to the standard i.m. dosing, using unadjuvanted RBD dosed i.m. or i.n. as a control. Doses of all components were identical between the i.m. and i.n. routes as outlined in **Table 1**. Study timeline was carried out as outlined in **Figure 1**. Serum, spleen, bone marrow, and BAL samples were collected on Day 42. Readouts were chosen to measure the effect of route and formulation on both systemic and mucosal immunogenicity. IgG2a and IgG1 were used as metrics of Th1 and Th2 immunity, respectively, and their ratio was used to characterize the induced immune phenotype (37, 38).

**Figure 2:**
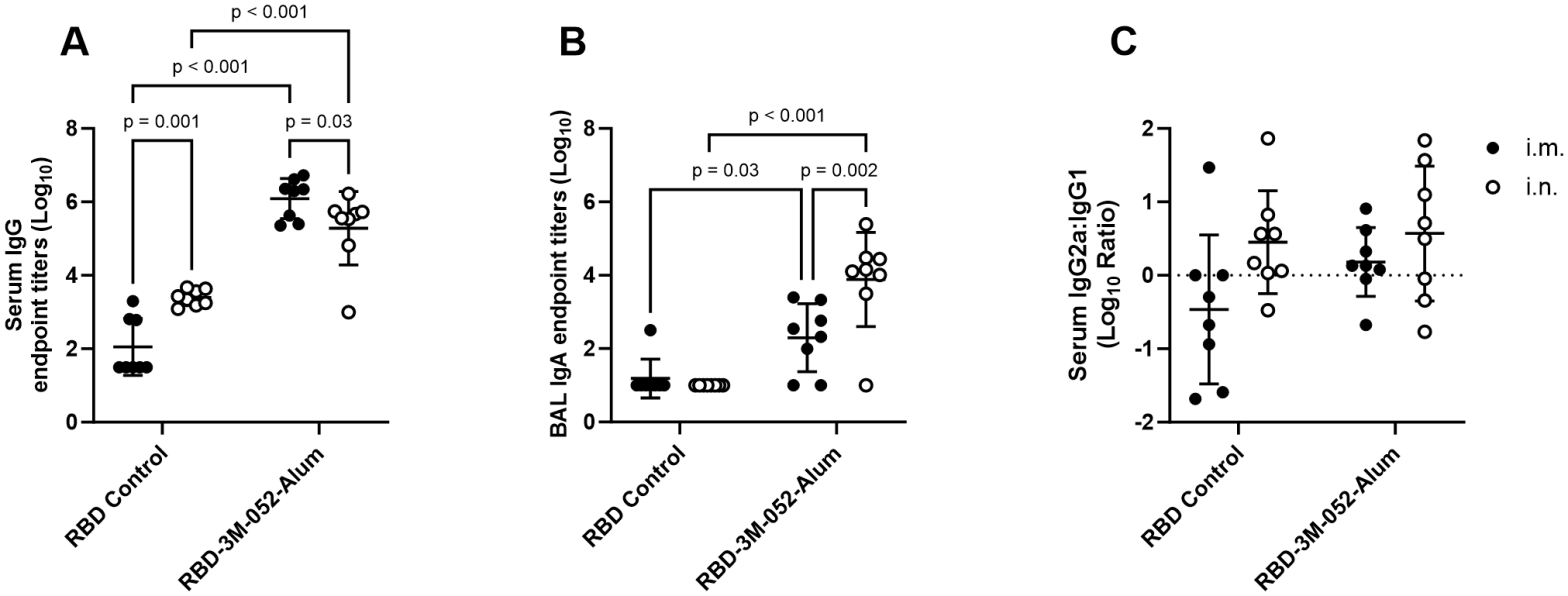
Route of delivery and adjuvant formulation affect antibody isotype class switching. **(A)** Serum titer of total anti-RBD IgG, **(B)** BAL titer of anti-RBD IgA, and **(C)** log_10_ transform of serum ratio of exponentiated anti-RBD IgG2a/IgG1 titers. Data collected from *n* = 8 (4M:4F) animals on Day 42 after being vaccinated twice intramuscularly (i.m.) or intranasally (i.n.) on Days 0 and 21 with RBD in combination with the 3M-052-Alum adjuvant, or without an adjuvant in the case of the unadjuvanted RBD control. The study was divided in half and vaccinations/harvests were staggered 1 week apart to reduce operator burden. Assays presented here were performed for all animals simultaneously using frozen serum and BAL samples. Horizontal bars represent the mean ± SD of log-normalized data. Statistical significance was determined via two-way ANOVA followed by Holm-Sidak’s correction for multiple comparisons, fixing the family-wide error rate to 0.05. Comparisons were made for the same adjuvant between i.m. and i.n. delivery, and between the adjuvanted and unadjuvanted RBD via each route of delivery (4 comparisons total).

Day 42 anti-RBD serum IgG titers (**Figure 2A**) were greater in mice who received the RBD-3M-052-Alum vaccine by 2-4 logs compared to unadjuvanted RBD, regardless of route of delivery (*p* < 0.001 for both comparisons). The unadjuvanted RBD induced greater serum anti-RBD IgG titers when dosed i.n. compared to i.m. (*p* < 0.001). Conversely, the i.m. dosed RBD-3M-052-Alum led to ~0.8 log higher serum IgG titers compared to the i.n. dosed vaccine, 6.09 ± 0.55 vs 5.28 ± 1.00 respectively (*p* = 0.03). In aggregate, observed differences in serum anti-RBD IgG were dominated by the choice of adjuvant but not significantly impacted by the route of delivery (*p* < 0.001 and *p* = 0.28, respectively, by two-way ANOVA).

IgA titers quantified in BAL samples were used as a measure of mucosal immunogenicity. Day 42 anti-RBD BAL IgA titers (**Figure 2B**) showed that the 3M-052-Alum adjuvant improved the response rate among animals regardless of route of delivery. 13 of 16 animals that received the 3M-052-Alum adjuvanted RBD vaccine either i.m. or i.n. generated a measurable BAL IgA response, whereas the unadjuvanted RBD elicited a measurable response in only 1 of 16 animals, via the i.m. route, with no i.n. response. Dosing the 3M-052-Alum adjuvanted RBD i.n. increased BAL IgA titers by ~1.5 logs compared to i.m. dosing, from 2.30 ± 0.93 to 3.88 ± 1.28 logs (*p* < 0.001). The RBD-3M-052-Alum vaccine led to higher BAL anti-RBD IgA titers compared to the unadjuvanted RBD vaccine via either i.n. or i.m. delivery (*p* ≤ 0.05 for all comparisons). Unlike serum IgG, both choice of adjuvant and route of delivery were significant sources of variation in BAL IgA secretion (*p* = 0.005 and *p* = 0.02, respectively, by two-way ANOVA).

The log_10_ ratio of exponentiated serum IgG2a to IgG1 titers was measured at Day 42 as an indication of the relative balance of Th1- and Th2-type immune responses (**Figure 2C**). There were no statistically significant differences detected between adjuvant groups or routes of delivery (*p* > 0.3 for all comparisons), and there was no detected effect of either adjuvant choice or route of delivery (*p* > 0.1 for both comparisons via two-way ANOVA). In summary, route of delivery influenced anti-RBD serum IgG and BAL IgA responses, but route of delivery had no detectable effect on Th1/Th2 balance.

**Figure 3:**
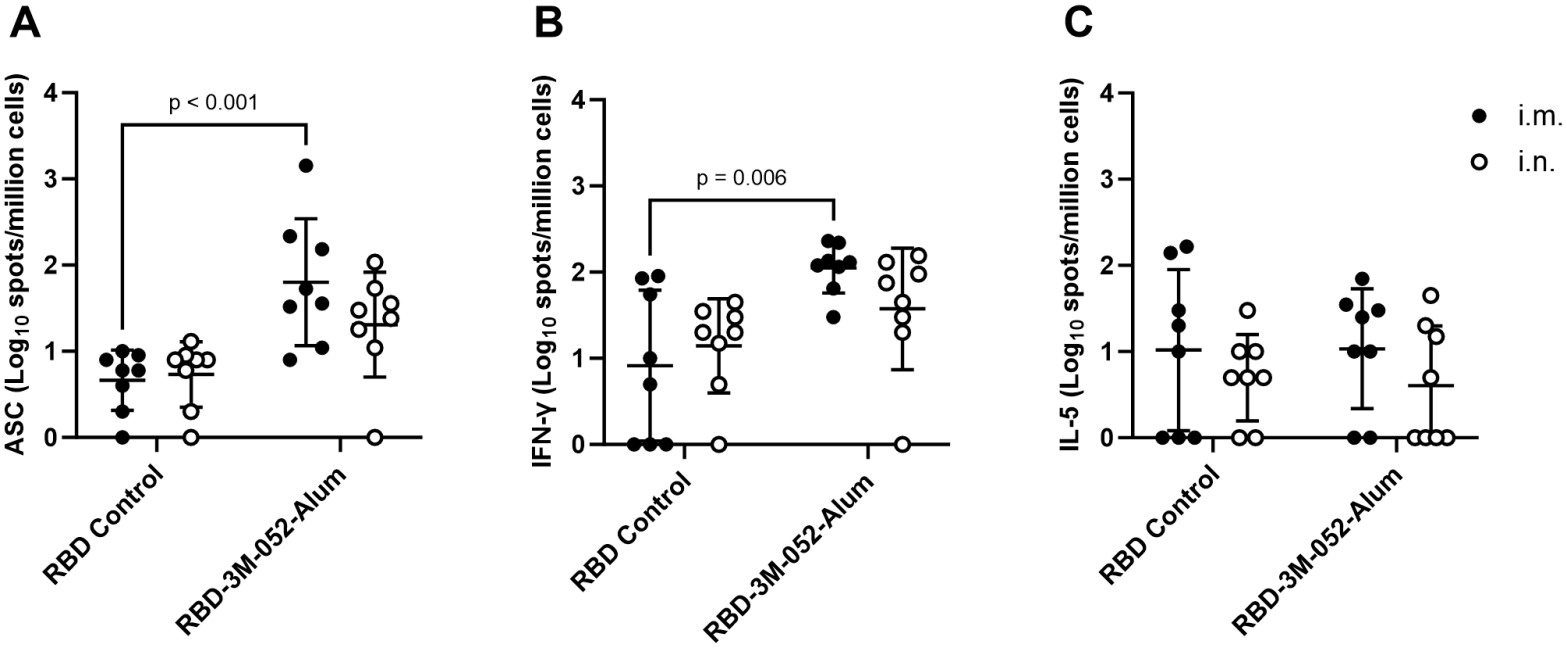
Route of delivery has minimal effect on bone marrow and splenocyte cellular responses to the RBD vaccine. **(A)** Bone marrow-derived anti-full-length-wt-Spike IgG antibody-secreting cells (ASC) ELISpot. T cell ELISpot measurement of splenocytes secreting **(B)** IFN-γ or **(C)** IL-5 upon stimulation with a SARS-CoV-2 peptide pool. Data collected from *n* = 8 (4M:4F) animals on Day 42 after being vaccinated twice intramuscularly (i.m.) or intranasally (i.n.) on Days 0 and 21 with RBD in combination with 3M-052-Alum (or without an adjuvant in the case of the unadjuvanted RBD control). The study was divided in half and vaccinations/harvests were staggered 1 week apart to reduce operator burden. Assays presented here were performed at the time of tissue harvest. Horizontal bars represent the mean ± SD of the log-transformed data. Statistical significance was determined via two-way ANOVA followed by Holm-Sidak’s correction for multiple comparisons fixing the family-wide error rate to 0.05. Comparisons were made for the same adjuvant between i.m. and i.n. delivery, and between the adjuvanted and unadjuvanted RBD via each route of delivery (4 comparisons total).

Bone marrow ELISpot assays were performed to measure the population of bone marrow-resident anti-full length wt Spike IgG-secreting cells as a metric of humoral memory (**Figure 3A**). For the tested vaccination groups, there was no statistically significant difference in bone marrow antibody-secreting cell (ASC) ELISpot responses based only on the route of delivery (*p* > 0.1 for both comparisons). RBD-3M-052-Alum delivered via the i.m. or i.n. route increased the anti-full length wt Spike bone marrow IgG response compared to the i.m. unadjuvanted RBD by 1.14 log (*p* < 0.001). Choice of adjuvant was a significant source of variation in the bone marrow anti-wt-Spike IgG response, whereas route of delivery was not (*p* < 0.001 and *p* > 0.2, respectively), suggesting that adjuvant choice dominated the expansion of bone marrow-resident antibody-secreting cell populations.

The antigen-specific activity of splenocyte populations was measured via cytokine ELISpot assays. These assays measured splenocyte secretion of IFN-γ (**Figure 3B**) and IL-5 (**Figure 3C**) after stimulation with a commercially available SARS-CoV-2 peptide pool as a surrogate measure of Th1- and Th2-type immune responses, respectively. For both IFN-γ and IL-5, there was no statistically significant difference within each vaccinated group based on the route of delivery (*p* > 0.4 or 0.7 for all comparisons, respectively). RBD-3M-052-Alum delivered via the i.m. route increased splenocyte IFN-γ ELISpot responses compared to unadjuvanted RBD by 1.13 log (*p* = 0.006). Interestingly, while choice of adjuvant formulation was a significant source of splenocyte IFN-γ response variation (*p* = 0.002 via two-way ANOVA), there was no statistically significant effect on splenocyte IL-5 ELISpot responses from either route of delivery or adjuvant formulation (*p* > 0.1 for both comparisons via two-way ANOVA). These results suggest that the route of delivery had a negligible effect on splenocyte responses and, as expected, the use of an adjuvant can affect splenocyte responses in a pro-Th1 manner. Based on its overall robust immunogenicity profile, and to compare equivalent routes of administration, the i.m. RBD-3M-052-Alum was selected for further study in a heterologous combination with a SARS-CoV-2 saRNA-NLC vaccine.

### Heterologous combination with an saRNA-NLC vaccine

Based on the widespread use of multiple COVID-19 vaccine technologies in many countries, it is critical to know how new COVID-19 vaccines will interact with the pre-existing immunity generated by other vaccine modalities. To this end, we explored a heterologous combination between the 3M-052-Alum adjuvanted RBD vaccine and a clinical-stage saRNA vaccine formulated in an NLC (31, 33), which was evaluated in a clinical trial against SARS-CoV-2 (NCT05370040), to determine if the order of administration affected the magnitude or phenotype of the immune response. As outlined in **Figure 1**, all animals were vaccinated i.m. on Days 0 and 21. Serum samples were collected on Days 21 and 42, and tissue samples were collected on Day 42. Animals were administered either two doses of the RBD-3M-052-Alum vaccine, two doses of the saRNA-NLC vaccine, or one dose of each in either order (see **Table 2**).

**Figure 4:**
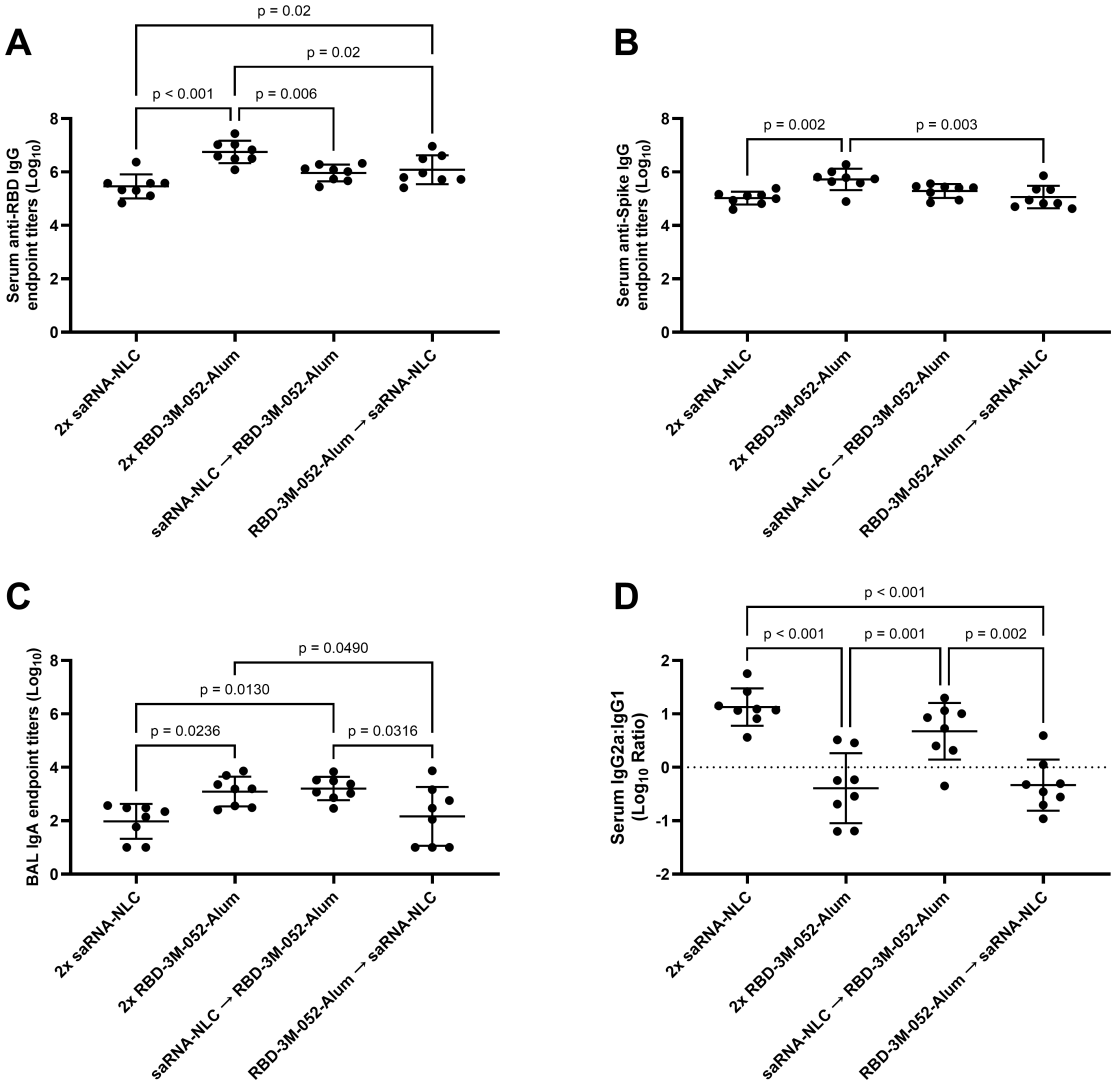
Vaccine regimen influences antibody titer and isotype class switching. **(A)** Serum titer of total anti-RBD IgG, **(B)** serum titer of total anti-full-length intact wt Spike IgG, **(C)** BAL titer of anti-RBD IgA, and **(D)** log_10_ transform of serum ratio of exponentiated anti-RBD IgG2a/IgG1 titers. Data were collected from *n* = 8 (4M:4F) animals on Day 42 after being vaccinated intramuscularly (i.m.) on Days 0 and 21 with the indicated vaccines in the indicated order. Arrow symbols demarcate heterologous prime-boost vaccinations (Prime → Boost). The study was divided in half and vaccinations/harvests were staggered 1 week apart to reduce operator burden. Assays presented here were performed for all animals simultaneously using frozen serum and BAL samples. Horizontal bars represent the mean ± SD of log-normalized data. Statistical significance was determined via one-way ANOVA followed by Holm-Sidak’s correction for multiple comparisons, fixing the family-wide error rate to 0.05.

The RBD-3M-052-Alum prime-boost regimen led to a >1 log higher Day 42 mean anti-RBD serum IgG titer compared to the other experimental regimens (*p* < 0.02 for all comparisons), and the RBD-3M-052-Alum prime-saRNA-NLC boost regimen led to a 0.6 log higher mean titer compared to the saRNA-NLC prime-boost regimen (**Figure 4A**). Since the saRNA antigen encodes the full-length SARS-CoV-2 Spike protein, we performed an otherwise identical ELISA using full-length intact recombinant wt Spike as the capture ligand to clarify if the observed difference in antibody titers was due to using RBD as a capture ligand (**Figure 4B**). The anti-Spike serum IgG titer induced by the RBD-3M-052-Alum prime-boost regimen was higher than the saRNA-NLC prime-boost regimen and the RBD-3M-052-Alum prime-saRNA-NLC boost regimen by ~0.7 log (*p* < 0.002 for both comparisons). This suggests that the observed difference in anti-RBD IgG results were not due to signal dilution by non-RBD binding sites in the case of the saRNA-NLC vaccine. There was no significant difference in serum anti-RBD IgG titers between regimen groups on Day 21 (**Supplementary Figure S1**) (*p* > 0.2 for all comparisons). The results in **Figures 4A, B** suggest that the RBD-3M-052-Alum vaccine prime-boost regimen had the strongest IgG response among the formulations and doses tested, regardless of the specific antigen; however, all regimens elicited very strong humoral responses approaching the upper limit of quantification for our assay.

Measurements of BAL anti-RBD IgA showed that the RBD-3M-052-Alum prime-boost and the saRNA-NLC prime-RBD-3M-052-Alum boost regimens both led to ~1 log higher BAL anti-RBD IgA titers than the saRNA-NLC prime-boost regimens and the RBD-3M-052-Alum prime-saRNA-NLC boost regimens (*p* < 0.05 for all comparisons) (**Figure 4C**). This suggests that boosting with the RBD-3M-052-Alum vaccine was important for generating mucosal IgA.

Ratios of the exponentiated titers of serum IgG2a and IgG1 were used as a metric of the relative Th1/Th2 balance of the induced immune response. The saRNA-NLC prime-boost and the saRNA-NLC prime-RBD-3M-052-Alum boost regimens increased mean serum IgG2a/IgG1 ratios compared to the RBD-3M-052-Alum prime-boost and the RBD-3M-052-Alum prime-saRNA-NLC boost regimens by 1-1.5 logs (*p* ≤ 0.002 for all comparisons). These results imply that priming with saRNA-NLC was correlated with increased serum IgG2a/IgG1 ratios, suggesting a Th1-skewed response. On the other hand, priming with RBD-3M-052-Alum led to a lower serum IgG2a/IgG1 ratio regardless of the boost dose, implying a more Th2-skewed response.

**Figure 5:**
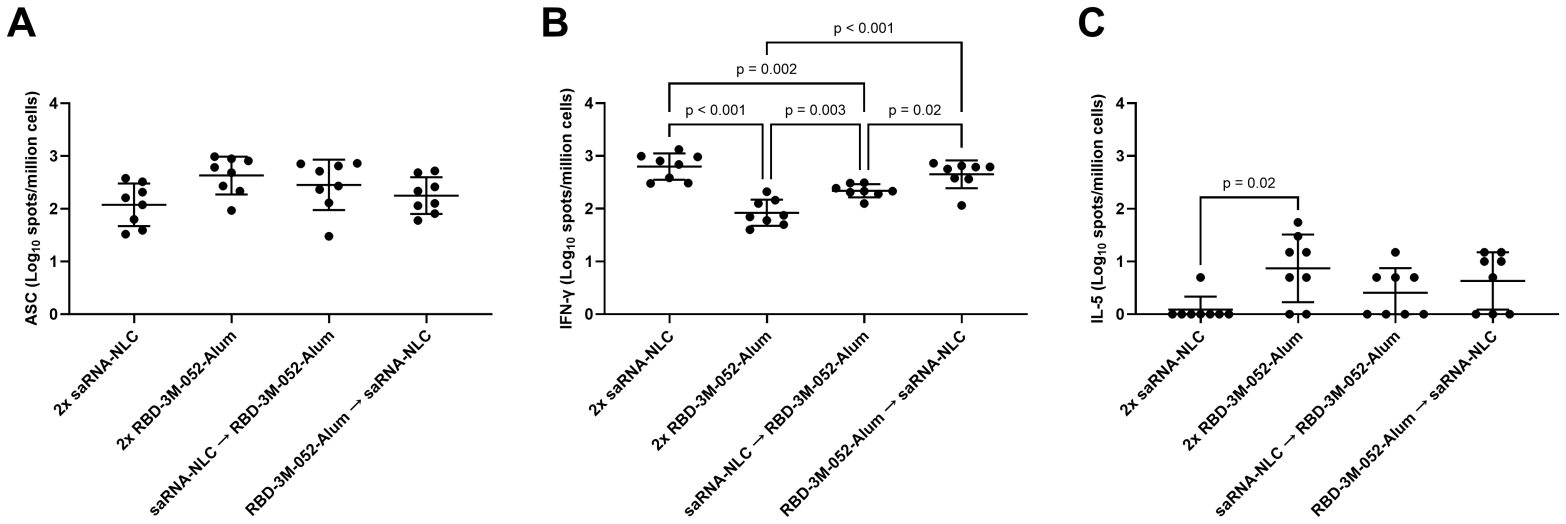
Vaccination regimen impacts cellular response phenotype. **(A)** Bone marrow-derived anti-full-length-wt-Spike IgG antibody-secreting cells (ASC) ELISpot. T cell ELISpot measurement of splenocytes secreting **(B)** IFN-γ or **(C)** IL-5 upon stimulation with a SARS-CoV-2 peptide pool. Data were collected from *n* = 8 (4M:4F) animals on Day 42 after being vaccinated intramuscularly (i.m.) on Days 0 and 21 with the indicated vaccines in the indicated order. Arrow symbols demarcate heterologous prime-boost vaccinations (Prime → Boost). The study was divided in half and vaccinations/harvests were staggered 1 week apart to reduce operator burden. Assays presented here were performed at the time of tissue harvest. Horizontal bars represent the mean ± SD of the log-transformed data. Statistical significance was determined via one-way ANOVA followed by Holm-Sidak’s correction for multiple comparisons fixing the family-wide error rate to 0.05.

ELISpot assays of bone marrow and splenocyte isolates were used to further investigate the cellular response to the heterologous combination of the saRNA-NLC and RBD-3M-052-Alum vaccines. There was no significant difference between regimens in terms of bone marrow-derived anti-Spike IgG-secreting cell populations (*p* > 0.06 for all comparisons, **Figure 5A**). The RBD-3M-052-Alum prime-boost regimen produced a mean response of ~0.6-log greater than the saRNA-NLC prime-boost regimen; however, this difference did not reach statistical significance (*p* = 0.0558). There was not a clear effect of vaccine regimen on bone marrow-resident antibody-secreting cell proliferation.

The saRNA-NLC prime-boost, RBD-3M-052-Alum prime-saRNA-NLC boost, and saRNA-NLC prime-RBD-3M-052-Alum boost regimens produced a 0.88-, 0.73-, and a 0.41-log greater splenocyte IFN-γ ELISpot response (**Figure 5B**), respectively, compared to the RBD-3M-052-Alum prime-boost regimen (*p* < 0.003 for all comparisons), while the saRNA-NLC prime-boost and RBD-3M-052-Alum prime-saRNA-NLC boost regimens produced a 0.46- and 0.31-log greater splenocyte IFN-γ ELISpot response, respectively, compared to the saRNA-NLC prime-RBD-3M-052-Alum boost regimen (*p* < 0.02 for both comparisons). This implies that boosting with the saRNA-NLC vaccine increased splenocyte IFN-γ responses and Th1 biasing, regardless of the prime product; however, further study would be required to confirm this.

Further, the saRNA-NLC prime-boost regimen decreased the splenocyte IL-5 ELISpot response (**Figure 5C**) by more than 0.87 log in comparison to the RBD-3M-052-Alum prime-boost regimen (*p* < 0.01), although several animals from all regimen groups had IL-5 ELISpot responses below the limit of detection. This suggests that the saRNA-NLC vaccine was very strongly Th1 cytokine biasing when used in a homologous regimen. Collectively, these results further demonstrate how the choice of vaccine regimen influences the Th1/Th2 balance of the resulting immune response.

**Figure 6:**
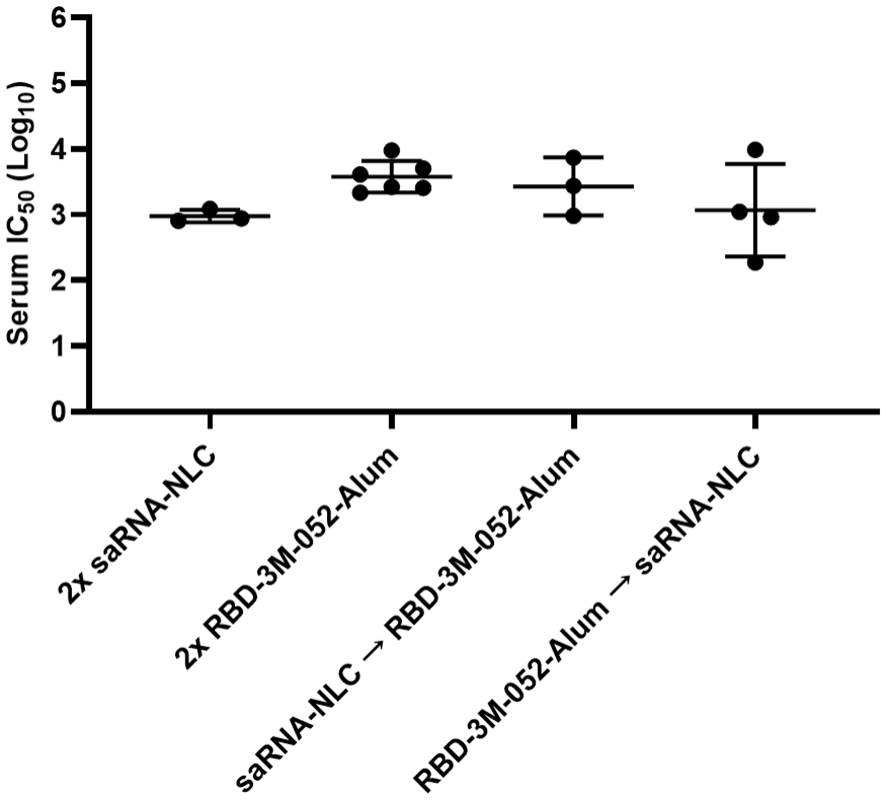
Pseudovirus neutralization is not significantly altered by heterologous regimens. Data were collected from *n* = 3-6 animals on Day 42 after being vaccinated intramuscularly (i.m.) on Days 0 and 21 with the indicated vaccines in the indicated order. Arrow symbols demarcate heterologous prime-boost vaccinations (Prime → Boost). The study was divided in half and vaccinations/harvests were staggered 1 week apart to reduce operator burden. Assays presented here were performed for all animals simultaneously using frozen serum samples. Day 42 serum samples were tested in a pseudovirus neutralization assay against a Wuhan (wt) pseudovirus. Horizontal bars represent the mean ± SD of log-normalized data. Statistical significance was determined via one-way ANOVA, followed by a Holm-Sidak’s correction for multiple comparisons, fixing the family-wide error rate to 0.05.

The serum neutralization response induced by the tested vaccination regimens was measured using a pseudovirus neutralization assay as previously described (31, 36) using a wt variant pseudovirus (Wuhan-Hu-1) (**Figure 6**). No significant differences were detected in serum neutralizing log IC_50_ between any of the experimental regimens (*p* > 0.3 for all comparisons), which suggests that all regimens were equally effective at generating neutralizing humoral responses. Assay restrictions and reagent limitations precluded analysis of all serum samples via the pseudovirus neutralizing assay. Where necessary, the samples tested in **Figure 6** were randomly selected from remaining Day 42 serum samples from the animals in **Figure 4**.

**Figure 7:**
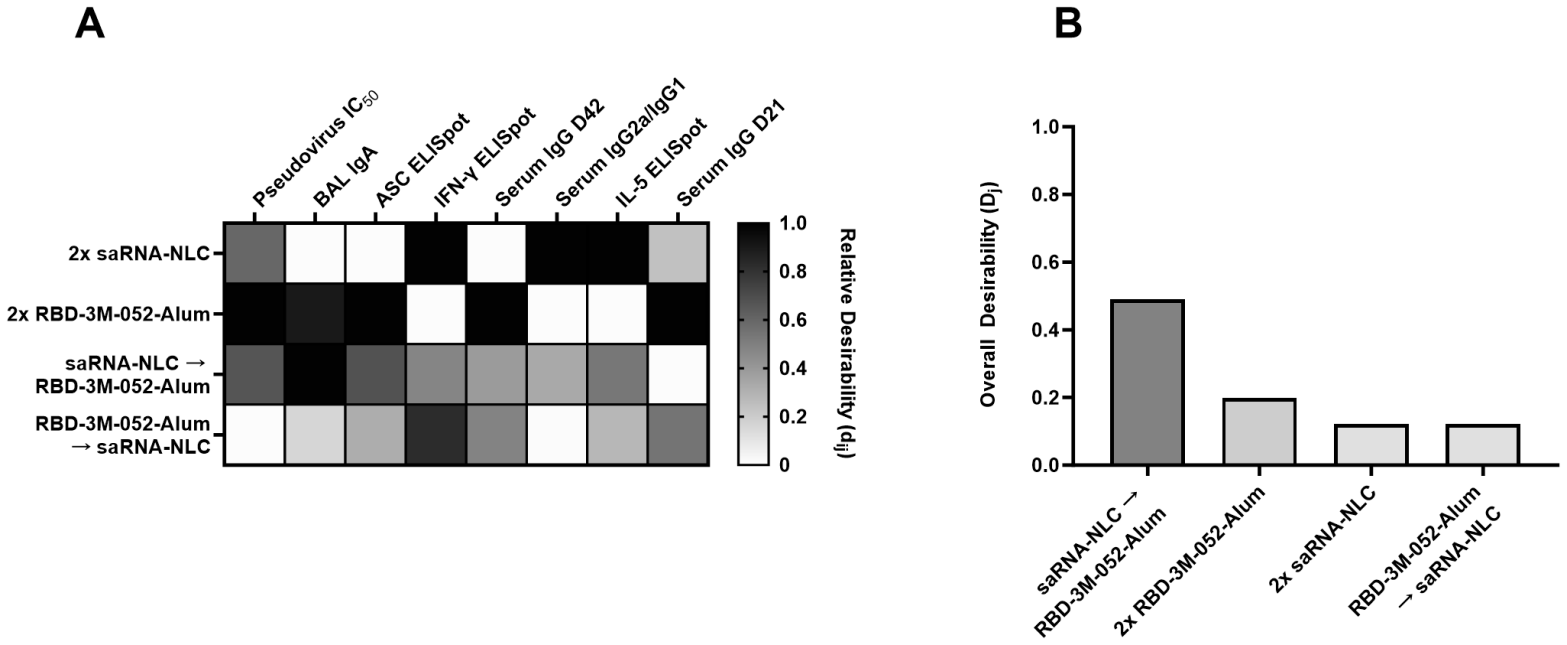
Identification of the most desirable dosing regimen. **(A)** Heatmap of individual desirability index scores (*d_ij_*) for the *j* ^th^ group and *i* ^th^ response. Columns are ordered left to right by decreasing weight (see **Supplementary Table S1**). Scores are normalized within each response variable. D = Day, BM = bone marrow, BAL = bronchoalveolar lavage. **(B)** Weighted, aggregate desirability scores (*D_j_*) per group (*j*), ordered left to right from highest to lowest score. The saRNA-NLC prime-RBD-3M-052-Alum boost and the RBD-3M-052-Alum prime-boost regimens were the most desirable based on the weights outlined in **Supplementary Table S1**. Desirability index results were calculated using formulas described in the **Supplementary Methods**. Arrow symbols demarcate heterologous prime-boost vaccinations (Prime → Boost).

Lead vaccine regimens were identified using a desirability index approach, see **Supplementary Methods** for a full process description. A desirability index allows for ranking multiple groups across a selected set of parameters by aggregating and normalizing those parameters using a pre-defined weighting scheme (39, 40). Weights and input factors are outlined in **Supplementary Table S1**. Weights were chosen to maximize readouts thought to be important for effective SARS-CoV-2 vaccines, such as serum pseudovirus neutralization, mucosal IgA secretion, bone marrow-resident antibody-secreting cell (ASC) populations, and Th1-response indicators (IgG2a/IgG1 ratio and splenocyte IFN-γ secretion) while minimizing Th2-response indicators (splenocyte IL-5 secretion) (41–43). Desirability responses broken down by factor can be seen in **Figure 7A**, and overall aggregate scores are shown in **Figure 7B**. The top two scoring regimens were the saRNA-NLC prime-RBD-3M-052-Alum boost (*D*_saRNA-NLC→RBD-3M-052-Alum_ = 0.491) and the RBD-3M-052-Alum prime-boost (*D*_2x-RBD-3M-052-Alum_ = 0.198) regimens, followed by the saRNA-NLC prime-boost (*D*_2x-saRNA-NLC_ = 0.121) and the RBD-3M-052-Alum prime-saRNA-NLC boost (*D*_RBD-3M-052-Alum→saRNA-NLC_ = 0.120) regimens. Therefore, the saRNA-NLC prime-RBD-3M-052-Alum boost and RBD-3M-052-Alum prime-boost regimens were selected for further investigation as freeze-dried preparations to determine the effect of lyophilization on immunogenicity and thermostability.

### Lyophilization and thermostability of RBD-3M-052-Alum

There is an acute need for vaccines that can be transported and stored at non-frozen temperatures. The saRNA-NLC vaccine has been previously shown to remain stable and immunogenic for more than 6 months when lyophilized and stored at room temperature, and more than 10 months when lyophilized and stored at refrigerated temperatures (31, 32). To see if a similar approach was feasible with the RBD-3M-052-Alum vaccine, we generated a proof-of-concept lyophilized formulation and tested the immunogenicity of the resulting material head-to-head with liquid vaccine preparations prepared immediately prior to use. Lyophilized formulations were prepared in a single-vial presentation, including 10-20% w/v sucrose as a lyoprotectant, and stored at 4°C for 30 days until use. On the day of vaccination, the lyophilized vials were reconstituted with Milli-Q water prior to administration.

This study was performed analogously to the study described in **Table 2** using the design outlined in **Figure 1**. Each animal received two vaccinations on Days 0 and 21 with tissue and serum harvested on Day 42. Liquid vaccine preparations were mixed on the day of administration, and lyophilized presentations of the saRNA-NLC and RBD-3M-052-Alum vaccines were reconstituted with Milli-Q water; both types were used within 1 h of preparation. Eight animals per group (4M:4F) received the homologous liquid or lyophilized RBD-3M-052-Alum prime-boost regimen, or the heterologous liquid or lyophilized saRNA-NLC prime-RBD-3M-052-Alum boost regimen.

**Figure 8:**
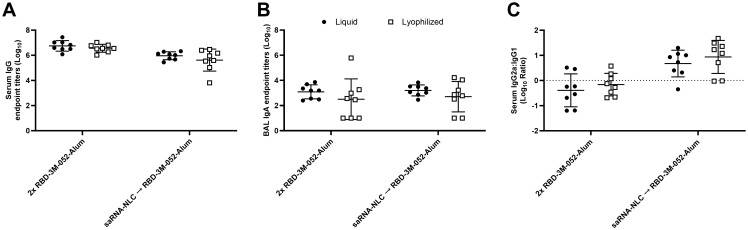
The lyophilization process does not significantly impact the humoral immune response. **(A)** Serum titer of total anti-RBD IgG, **(B)** BAL titer of anti-RBD IgA, and **(C)** ratio of exponentiated serum anti-RBD IgG2a/IgG1 titers. Data were collected from *n* = 8 (4M:4F) animals on Day 42 after being vaccinated twice intramuscularly (i.m.) on Days 0 and 21 with the indicated vaccines in the indicated order. Arrow symbols demarcate heterologous prime-boost vaccinations (Prime → Boost). The study was divided in half and vaccinations/harvests were staggered 1 week apart to reduce operator burden. Assays presented here were performed for all animals simultaneously using frozen serum and BAL samples. Horizontal bars represent the mean ± SD of log-normalized data. Statistical significance was determined via two-way ANOVA followed by Holm-Sidak’s correction for multiple comparisons, fixing the family-wide error rate to 0.05. The study was performed simultaneously with **Figure 4** and **Figure 5**; liquid material data are presented in both figures.

No significant differences were observed between the liquid and lyophilized vaccine preparations in terms of serum anti-RBD IgG titer (**Figure 8A**), BAL anti-RBD IgA titer (**Figure 8B**), or serum IgG2a/IgG1 ratio (**Figure 8C**) 42 days post-prime (*p* > 0.08 for all comparisons). Similarly, no differences were observed between the liquid and lyophilized vaccine preparations in terms of the measured ELISpot responses: bone marrow-derived anti-Spike IgG-secreting cells and splenocyte-derived IFN-γ- or IL-5-secreting cells (*p* > 0.3 for all comparisons) (**Supplementary Figures S2A–C**). This suggests that there was no difference in immunogenicity between the lyophilized and liquid presentations of the RBD-3M-052-Alum or saRNA-NLC products. The lyophilized vaccine groups had greater number of non-responders in the BAL IgA assay (**Figure 8B**) in both the RBD-3M-052-Alum prime-boost and the saRNA-NLC prime RBD-3M-052-Alum boost regimens (3-out-of-8 and 2-out-of-8 non-responders, respectively). Further study is necessary to understand if this is an artifact or related to the vaccine lyophilization process.

As a metric of its suitability for lyophilization and high temperature stability, the physical stability of the lyophilized RBD-3M-052-Alum vaccine composition was evaluated before and after lyophilization, and after storage at elevated temperatures (**Supplementary Figure S3**). Particle size was not significantly changed by lyophilization (**Supplementary Figure S3A**) or after storage of the lyophilized material for 4 weeks at 4°C or 40°C, or 6 weeks at 25°C (**Supplementary Figure S3B**). The melting temperature (Tm) of the RBD adsorbed onto the surface of the Alum was assessed using differential scanning fluorimetry by measuring the ratio of fluorescence emission at 330/350 nm with excitation at 295 nm. The Tm of the RBD dropped by 0.4 ± 0.06°C post lyophilization (*p* = 0.002) (**Supplementary Figure S3C**). After storage of the post-lyophilization material at 4°C for 4 weeks there was no change in Tm compared to the initial post-lyophilization material (*p* = 0.49) (**Supplementary Figure S3D**). However, after storage of the lyophilized material at 25°C for 6 weeks or 40°C for 4 weeks, the Tm of the lyophilized product decreased by 2.7°C and 3.2°C, respectively, compared to the initial post-lyophilization material (*p* < 0.001 for all comparisons). In summary, these results demonstrate that the RBD-3M-052-Alum composition can be lyophilized without detrimental effects on its physical properties for at least 4 weeks at 4°C, although additional development may be required to optimize its thermostability profile at higher temperatures and/or for longer durations.

## Discussion

Generating effective protective responses in the respiratory tract is a critical factor in preventing the spread of SARS-CoV-2 and other respiratory pathogens (12). The gold standard metric that correlates with an effective mucosal response is the generation of mucosally secreted antigen-specific IgA, which plays a key role in defending mucosal surfaces in the sinus, respiratory, digestive, and urogenital tracts (44, 45). Here, we showed that the route of administration of an RBD-3M-052-Alum vaccine impacts several aspects of the immune response, as expected based on previous literature (46, 47). For instance, dosing the RBD-3M-052-Alum vaccine i.n. led to a greater BAL IgA response compared to the i.m. regimen, but a reduced serum IgG response (**Figures 2A, B**). However, there was no significant difference between i.n. and i.m. dosing of the RBD-3M-052-Alum vaccine in terms of elicited bone marrow-resident antibody-secreting cell populations (**Figure 3A**), or Th1- vs Th2-type biasing, as measured by serum IgG2a/IgG1 ratio (**Figure 2C**) and splenocyte IFN-γ or IL-5 ELISpot (**Figures 3B–C**). This suggests that the route of administration of the RBD-3M-052-Alum vaccine primarily influences the characteristics of the humoral response as opposed to altering the magnitude or phenotype of cellular responses. It is presently unknown if these responses could be further optimized by combining or alternating i.m. and i.n. dosing to achieve higher titers of both mucosal IgA and serum IgG, as has been demonstrated for other experimental subunit vaccines (6, 48, 49). Additionally, the i.m. and i.n. formulations used in this study were identical, so approaches to increase the transmembrane absorption of the vaccine formulation via permeation enhancers or receptor targeting might yield improved responses (50, 51).

The availability of many new vaccines in response to the SARS-CoV-2 pandemic has created a need to understand the immunological implications of heterologous vaccine regimens. A number of clinical trials have now established that distinct vaccine types can be used interchangeably in prime-boost dosing regimens to form productive immune responses against SARS-CoV-2 (16, 52). Many clinical studies have shown that the *Spikevax* (Moderna) and *Comirnaty* (Pfizer/BioNTech) vaccines, which both use base-modified mRNA encoding the SARS-CoV-2 Spike protein delivered via a lipid nanoparticle, can be interchanged without loss in protection (16, 17). However, some studies have shown a marked reduction in immunogenicity when patients receive an adjuvanted subunit vaccine, such as *Nuvaxovid* (Novavax), as a booster following an mRNA vaccine, but it is unknown if this is due to the specific vaccine itself or the nature of adjuvanted subunit vaccines in general (17). In our study of heterologous vaccine prime-boost combinations, we found that both the RBD-3M-052-Alum vaccine and the saRNA-NLC vaccine generally led to strong humoral responses, measured in terms of serum IgG titers (**Figures 4A, B**) and BAL IgA titers (**Figure 4C**); both of which were induced more strongly by the RBD-3M-052-Alum vaccine. In comparison, the saRNA-NLC vaccine led to a more Th1-skewing phenotype, as indicated by increased IgG2a/IgG1 ratios (**Figure 4D**) and splenocyte IFN-γ responses (**Figure 5B**). These benefits also extended to heterologous prime-boost regimens that included doses of the RBD-3M-052-Alum or saRNA-NLC vaccine. Notably, the saRNA-NLC prime-RBD-3M-052-Alum boost regimen led to a significantly higher BAL IgA response than the saRNA-NLC prime-boost regimen, and a significantly higher splenocyte IFN-γ response than the RBD-3M-052-Alum prime-boost regimen, suggesting that this regimen benefits from both vaccine modalities. The identification of idealized regimens was performed using a desirability index approach (**Figure 7**), and for the selected optimization factor weights (**Supplementary Table S1**), the most desirable regimen was saRNA-NLC prime-RBD-3M-052-Alum boost, largely due to its strong BAL IgA and Th1-biased responses. In contrast, the RBD-3M-052-Alum prime-saRNA-NLC boost regimen scored the lowest in our desirability index and did not generate the same benefits in terms of BAL IgA response or serum IgG2a/IgG1 ratio as the opposite regimen, suggesting that both the composition and the order of administration were both strong contributors to the overall immune phenotype. Thus, heterologous vaccine regimens benefit from the optimization of practical aspects, such as order of administration, which can significantly influence the response magnitude and phenotype. This approach has utility in the development of future vaccination schedules where rationally designed vaccination regimens composed of two or more distinct drug products are the final clinical deliverable, similar to modern combination immune-oncology therapies (53, 54).

A lyophilized form of the RBD-3M-052-Alum vaccine was prepared as a proof-of-concept thermostabilized composition. For vaccines that are designed for use in low- and middle-income countries, or any geographic region without easy access to cryogenic storage or refrigerated cold-chain transport, elevated temperature stability is often a critical design goal (2, 3, 55). Preparations of thermostabilized biologics often use lyophilization or spray drying as a means to preserve the bioactivity of the active pharmaceutical ingredients, and the success of these approaches has been demonstrated in single-vial preparations of adjuvanted protein subunit vaccines (31, 56–58). Our results showed no loss in immunogenicity of the RBD-3M-052-Alum vaccine after lyophilization whether used in a homologous prime-boost or in combination with a previously optimized lyophilized form of the saRNA-NLC vaccine (31, 32) (**Figure 8**; **Supplementary Figure S2**). Further work will be needed to optimize the thermostability profile of the RBD-3M-052-Alum composition and demonstrate long-term storage stability at non-refrigerated temperatures.

There are a few notable limitations in the interpretation of the heterologous vaccination results. First, in the studies presented here, the dosing and sampling interval was the same for each experimental regimen. Recent results from studies in mice (59) and clinical trials (60) have shown that the optimal interval between the prime and boost doses of a SARS-CoV-2 mRNA vaccine is likely 8-10 weeks or longer. The optimal dosing interval for adjuvanted subunit vaccines is likely highly dependent on the specific adjuvant and antigen of interest; however, some clinical-stage SARS-CoV-2 adjuvanted subunit vaccines have demonstrated similar enhancement using longer dosing intervals (61). It is possible that extending the dosing interval used in this study from 3 weeks (21 days) to 8 weeks or longer would have impacted the immunogenicity outcomes and could have altered the desirability ranking. There may be value in repeating these experiments either with larger group sizes or with different mouse strains to verify the reproducibility of these results. We have previously investigated the use of the RBD203-N1 antigen with the 3M-052-Alum adjuvant in an identical animal model, and we observed similar trends and results (29). Additionally, in our study of heterologous regimens, all regimens led to statistically equivalent neutralizing antibody responses; however, this may not be indicative of the response of a given regimen in a viral challenge study. There is also interest in the characterization of CD8^+^ and other effector function-specific T cell subsets in understanding the implications of different vaccine modalities (62); however, those analyses were beyond the scope of the present study.

In conclusion, we show that the route of delivery for adjuvanted subunit vaccines significantly influences the immune response phenotype, and that the combination of adjuvanted subunit and saRNA vaccine platforms in heterologous prime-boost regimens may offer immunological benefits. Moreover, immunogenicity profiles of the RBD-3M-052-Alum vaccine can be maintained following lyophilization, potentially enabling global distribution and storage. We believe these results are especially relevant in the continuously evolving vaccine ecosystem, where heterologous combinations of multiple vaccine brands and modalities have become the norm and where the need for stabilized vaccine preparations continues to grow (3, 63).

## Data Availability

The raw data supporting the conclusions of this article will be made available by the authors, without undue reservation.
