# Supplementary material for: Optimizing immunogenicity and product presentation of a SARS-CoV-2 subunit vaccine composition: effects of delivery route, heterologous regimens with self-amplifying RNA vaccines, and lyophilization
Source: Front Immunol. 2024 Dec 16;15:1480976. doi: 10.3389/fimmu.2024.1480976 (PMC11683073; doi:10.3389/fimmu.2024.1480976)
Supplement: Supplementary file 1 [file DataSheet1.docx]

# Supplementary Methods

## Desirability Index Calculations

Replicate data from each group were condensed into a descriptive statistic, using the geometric mean for all samples. Each group was then assigned a score for each parameter via Eqn. 1 to maximize a parameter or Eqn. 2 to minimize a parameter.

Where $d_{ij}$ is the desirability of the *i*^th^ parameter of group *j* and $\mu_{ij}$ is the descriptive statistic of the *i*^th^ parameter of group *j*, ${UB}_{i}$ (upper bound of parameter *i*) and ${LB}_{i}$ (lower bound of parameter *i*) were calculated using Eqn. 3 and Eqn. 4, respectively, where $\mu_{i:}$ is a vector containing all descriptive statistics for parameter *i*. Once all $d_{ij}$ were known, the aggregate desirability of group *j*, $D_{j}$, was calculated using Eqn. 5, where $p$ is the number of parameters and $w_{i}$ is the assigned weight of parameter *i* (Supplementary Table S1).

| Eqn. 5 | $D_{j}=\left( \prod_{1}^{p} d_{ij}^{w_{i}} \right)^{\frac{1}{\sum_{1}^{p} w_{i}}}$ |  |
| --- | --- | --- |

| Eqn. 3 | ${UB}_{i}=max\left( \mu_{i:} \right)+1\%\times\left( max\left( \mu_{i:} \right)-min\left( \mu_{i:} \right) \right)$ |  |
| --- | --- | --- |
| Eqn. 4 | ${LB}_{i}=min\left( \mu_{i:} \right)-1\%\times\left( max\left( \mu_{i:} \right)-min\left( \mu_{i:} \right) \right)$ |  |

| Eqn. 1 | $d_{ij}=\frac{\mu_{ij}-LB_{i}}{{UB}_{i}-{LB}_{i}}$ |  |
| --- | --- | --- |
| Eqn. 2 | $d_{ij}=\frac{{UB}_{i}-\mu_{ij}}{{UB}_{i}-{LB}_{i}}$ |  |

# Supplementary Data

**Supplementary Table S1**: Desirability index weights and parameters

| **Parameter** | **Unit** | **Weight** | **Optimization Goal** |
| --- | --- | --- | --- |
| Pseudovirus Neutralization | IC_50_ (Log_10_) | 5 | Maximize |
| BAL anti-RBD IgA | Titer (Log_10_) | 4 | Maximize |
| Bone marrow IgG ELISpot | Spots/Million Cells (Log_10_) | 4 | Maximize |
| IFN-γ ELISpot | Spots/Million Cells (Log_10_) | 3 | Maximize |
| IL-5 ELISpot | Spots/Million Cells (Log_10_) | 3 | Minimize |
| Serum anti-RBD IgG Day 42 | Titer (Log_10_) | 3 | Maximize |
| Serum IgG2a/IgG1 Day 42 | Ratio of exponentiated titers | 3 | Maximize |
| Serum anti-RBD IgG Day 21 | Titer (Log_10_) | 1 | Maximize |


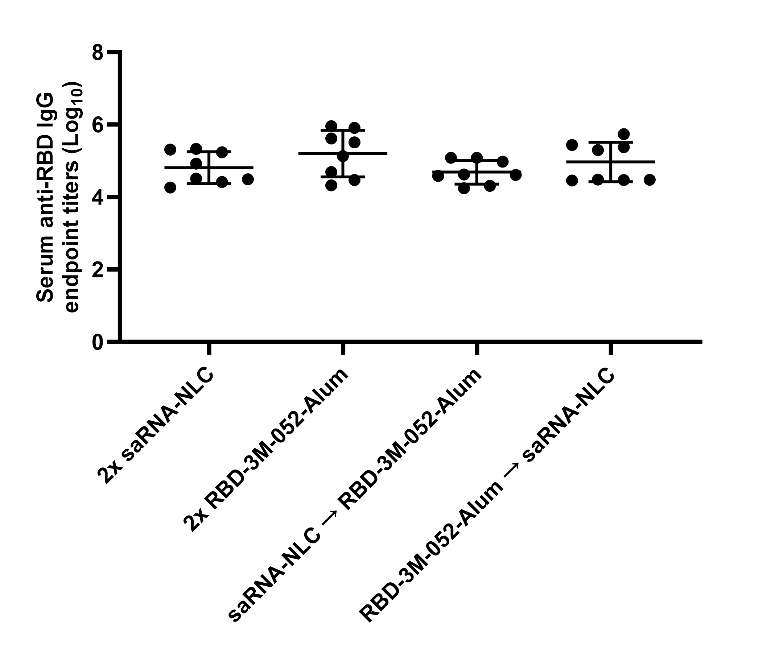


**Supplementary Figure S1:** No difference in Day 21 serum anti-RBD IgG titer between heterologous groups. Data collected from *n* = 8 (4M:4F) animals on Day 21 after being vaccinated intramuscularly (i.m.) on Day 0 with the indicated vaccines. Arrow symbols demarcate heterologous prime-boost vaccinations (Prime → Boost). For heterologous groups, both vaccines have been listed for clarity, but animals have only received the boost dose at the time of serum collection. The study was divided in half and vaccinations/harvests were staggered 1 week apart to reduce operator burden. Assays presented here were performed for all animals simultaneously using frozen serum samples. Statistical significance was determined via one-way ANOVA followed by Holm-Sidak’s correction for multiple comparisons, fixing the family-wide error rate to 0.05. Horizontal bars represent the mean ± SD of log-normalized data.


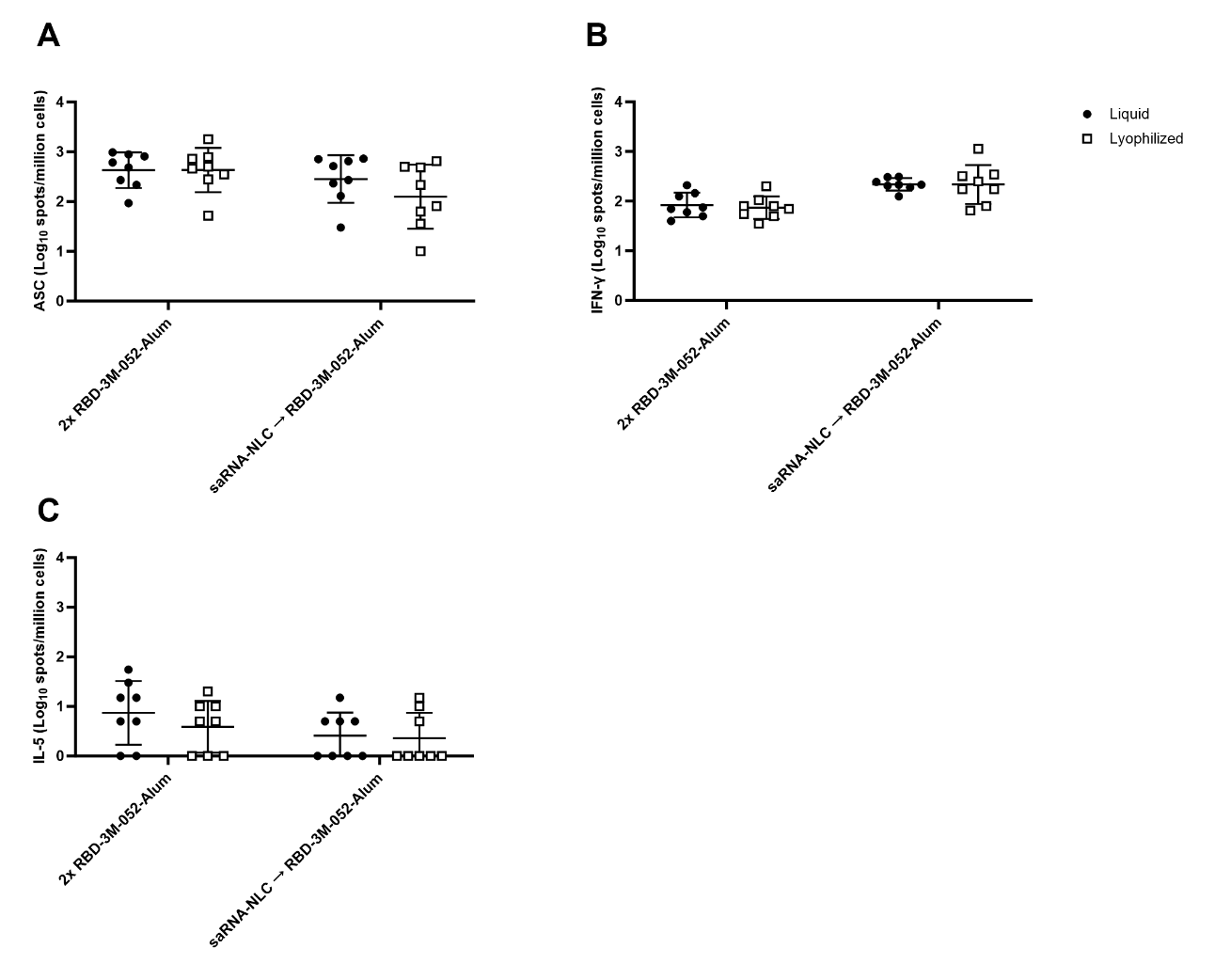


**Supplementary Figure S2:** Lyophilization process does not impact cellular response. (A) Bone marrow-derived anti-full-length-wt-Spike IgG antibody-secreting cells (ASC) ELISpot. T cell ELISpot measurement of splenocytes secreting (B) IFN-γ or (C) IL-5 upon stimulation with a SARS-CoV-2 peptide pool. Data collected from *n* = 8 (4M:4F) animals on Day 42 after being vaccinated twice intramuscularly (i.m.) on Days 0 and 21 with the indicated vaccines in the indicated order. Arrow symbols demarcate heterologous prime-boost vaccinations (Prime → Boost). The study was divided in half and vaccinations/harvests were staggered 1 week apart to reduce operator burden. Assays presented here were performed at the time of tissue harvest. Horizontal bars represent the mean ± SD of the log-transformed data. Statistical significance was determined via two-way ANOVA followed by Holm-Sidak’s correction for multiple comparisons fixing the family-wide error rate to 0.05. Note: study performed simultaneously with Figures 3 and 4; liquid material data are presented in both figures.


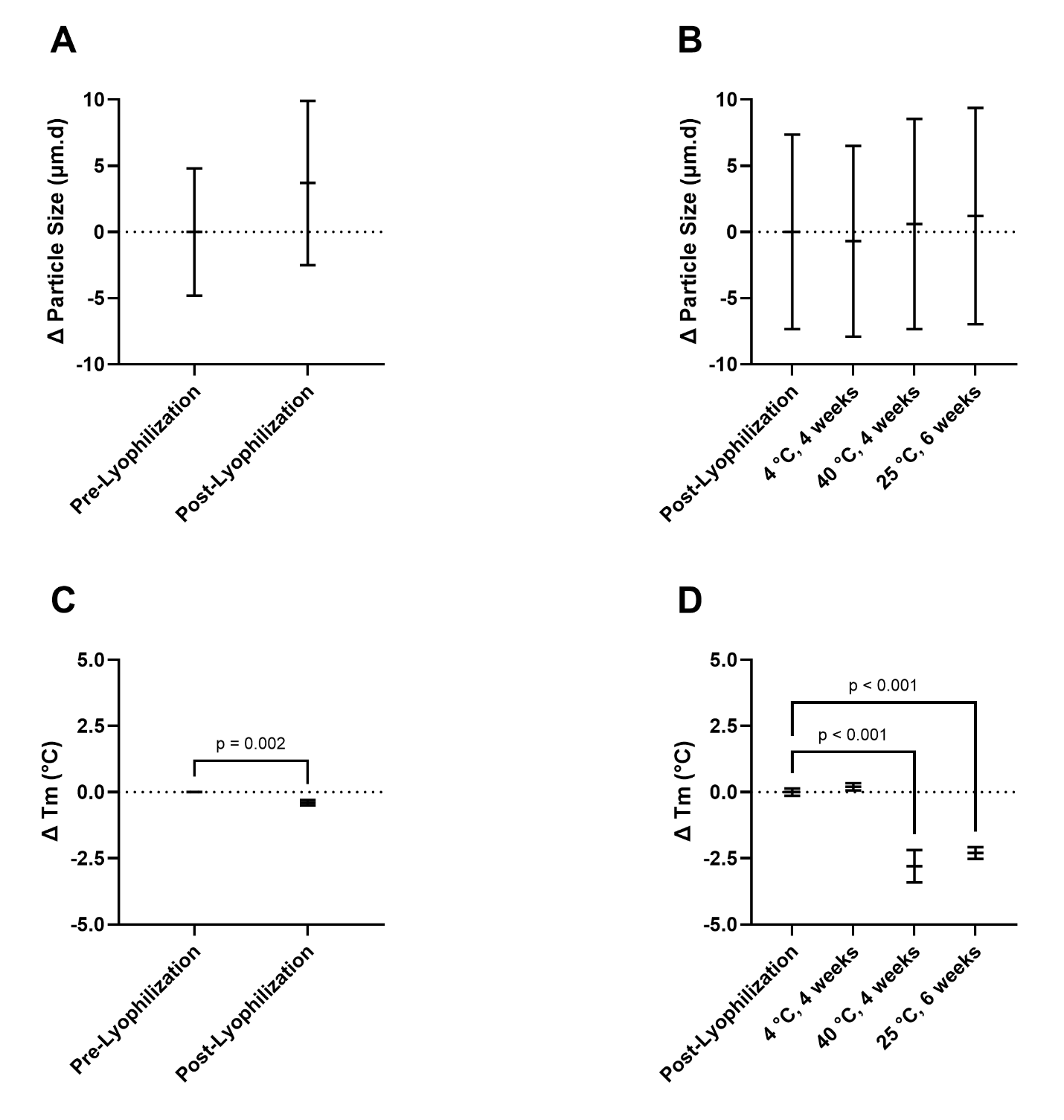


**Supplementary Figure S3:** Lyophilized RBD-3M-052-Alum vaccine maintains physical stability for at least 1 month at 4 °C. (A) Change (Δ) in particle size post-lyophilization, (B) change in particle size of lyophilized product after storage at the indicated conditions, (C) change in melting temperature post-lyophilization, and (D) change in melting temperature of lyophilized product after storage at the indicated conditions (statistical significance only calculated to Post-Lyophilization sample). Changes are given relative to the mean of the left most bar, propagating measurement error appropriately. Horizontal bars represent the mean ± SD of *n* = 3 replicates for each measurement. Statistical significance was determined via one-way ANOVA followed by Holm-Sidak’s correction for multiple comparisons fixing the family-wide error rate to 0.05. Note: in (C) and (D), error bars are plotted for all samples but may not be visible.
